# The *In Vitro* Biotransformation of the Fusion Protein Tetranectin-Apolipoprotein A1

**DOI:** 10.1038/s41598-019-40542-5

**Published:** 2019-03-11

**Authors:** Simone Schadt, Christophe Husser, Roland F. Staack, Aynur Ekiciler, Na Hong Qiu, Stephen Fowler, Christoph Funk, Nicole A. Kratochwil

**Affiliations:** 1 Roche Pharma Research and Early Development, Roche Innovation Center Basel, F. Hoffmann-La Roche Ltd, Grenzacherstr. 124, CH-4070 Basel, Switzerland; 2 Roche Pharma Research and Early Development, Roche Innovation Center Munich, Roche Diagnostics GmbH, Nonnenwald 2, 82377 Penzberg, Germany

## Abstract

As more and more protein biotherapeutics enter the drug discovery pipelines, there is an increasing interest in tools for mechanistic drug metabolism investigations of biologics in order to identify and prioritize the most promising candidates. Understanding or even predicting the *in vivo* clearance of biologics and to support translational pharmacokinetic modeling activities is essential, however there is a lack of effective and validated *in vitro* cellular tools. Although different mechanisms have to be adressed in the context of biologics disposition, the scope is not comparable to the nowadays widely established tools for early characterization of small molecule disposition. Here, we describe a biotransformation study of the fusion protein tetranectin apolipoprotein A1 by cellular systems. The *in vivo* biotransformation of tetranectin apolipoprotein A1 has been described previously, and the same major biotransformation product could also be detected *in vitro*, by a targeted and highly sensitive detection method based on chymotrypsin digest. In addition, the protease responsible for the formation of this biotransformation product could be elucidated to be DPP4. To our knowledge, this is one of the first reports of an *in vitro* biotransformation study by cells of a therapeutic protein.

## Introduction

Protein biotherapeutics are a class of pharmaceutical drugs with increasing importance for the treatment of various diseases. As more and more protein biotherapeutics enter the drug discovery pipelines, there is an increasing interest in tools for mechanistic drug metabolism and pharmacokinetics investigations of biologics in order to identify and prioritize the most promising candidates. Understanding or even predicting the *in vivo* clearance of biologics and to support translational pharmacokinetic modeling activities is essential, however there is a lack of effective and validated *in vitro* cellular tools^1^. While for small molecules, metabolic properties can be easily assessed *in vitro* using liver microsomes or hepatocytes, for protein biotherapeutics, the situation is far more complex. Protein biotherapeutics are biotransformed by proteases, and typically this degradation of the biomolecule yields no fragments of further biological relevance^2,3^. This catabolism by peptidases is an integral part of renal and hepatic clearance of proteins – typically via lysosomal degradation of the proteins to amino acids by cathepsins – and these processes do not result in circulating biotransformation products^2^. On the other hand, specific biotransformations also exist, resulting in measurable (and potentially bioactive) modified proteins. This type of biotransformation is often observed in fusion proteins, which are gaining popularity in pharmaceutical industry development pipelines^3,4^. In addition to proteolytic cleavages, deamidation, oxidation, or other amino acid modifications can alter the function of therapeutic proteins^5–9^. There are numerous endo- and exopeptidases found both in blood and endothelial cells^10^ capable of generating circulating biotransformation products. One example is Dipeptidyl-peptidase IV; DPP4 (Exopeptidase 3.4.14.5), which is expressed in endothelial cells, including kidney, liver, intestinal brush-border membranes, as well as throughout the capillary endothelium, and also a soluble form exists^11^. DPP4 cleaves dipeptides from peptides or proteins with the terminal structure motifs Xaa-Pro-Xaa or Xaa-Ala-Xaa at the N-terminus^12^. Prominent endogenous peptide substrates for DPP4 include the incretin hormones glucose-dependent insulinotropic polypeptide (GIP) and glucagon-like peptide-1 (GLP-1). DPP4 plays an important role in the regulation of both peptides, and inactivates them by N-terminal cleavage. Both GIP and GLP-1 have very short apparent plasma half-lives of 7 min and 1–2 min, respectively^11^. Degradation of GIP and GLP-1 cannot be assigned to one single tissue, but rather takes place in different tissues expressing DPP4^11^.

While a number of reports on *in vitro* biotransformation studies of GIP, GLP-1, and other peptides exist^13–15^, to date, there are only few, if any, reports on the *in vitro* biotransformation of therapeutic proteins using cellular systems. In this article, we describe the investigation and application of *in vitro* cellular systems for the biotransformation of the therapeutic fusion protein tetranectin apolipoprotein A1 (TN-ApoA1). This molecule is composed of a portion of human apolipoprotein A1 (ApoA1) and tetranectin (TN). The *in vivo* biotransformation of lipidated TN-ApoA1 has been described previously^16^. The formation of the major catabolite [3-285] of TN-ApoA1, generated by cleavage of AP at the N-terminus of the sequence, was fast and complete *in vivo* within 24 h after intravenous administration of TN-ApoA1 to rabbits and also in Cynomolgus monkeys and rats.

## Experimental Section

### Reference compounds

Lipidated TN-ApoA1 was prepared as follows: TN-ApoA1 was lipidated with pure POPC/DPPC (3/1; w/w) in a protein to lipid ratio of 1/60. POPC: Palmitoyl oleoyl phosphatidylcholine; DPPC: Dipalmitoyl phosphatidylcholine. The stock solution of lipidated TN-ApoA1 (10 mg/mL was prepared by dissolving the appropriate amount in aqueous solution of 250 mM TRIS buffer (pH 7.5), 10 mM methionine and 140 mM NaCl. The purity of TN-ApoA1 was >85%. The amino acid sequence of TN-ApoA1 is as follows:

APIVNAKKDVVNTKMFEELKSRLDTLAQEVALLKEQQALQTVDEPPQSPWDRVKDLATVYVDVLKDSGRDYVSQFEGSALGKQLNLKLLDNWDSVTSTFSKLREQLGPVTQEFWDNLEKETEGLRQEMSKDLEEVKAKVQPYLDDFQKKWQEEMELYRQKVEPLRAELQEGARQKLHELQEKLSPLGEEMRDRARAHVDALRTHLAPYSDELRQRLAARLEALKENGGARLAEYHAKATEHLSTLSEKAKPALEDLRQGLLPVLESFKVSFLSALEEYTKKLNTQ.

### Chemicals, Reagents and Materials

Formic acid, 98–100% (Suprapur) and ethanol (Lichrosolv) were purchased from Merck (Darmstadt, Germany). Acetonitrile (HPLC grade S) was obtained from Rathburn (Walkerburn, U.K.). Water was purified ‘in-house’ using a Milli-Q^plus^ 185 system from Millipore (Volketswil, Switzerland) to obtain de-ionized water. Water used for chromatography was Lichrosolv grade from Merck (Darmstadt, Germany). The 0.1% formic acid solution was prepared by adding 1.0 mL of formic acid (98–100%) to water in a 1.0-L volumetric flask and made up to volume with de-ionized water. Aqueous solution of Tween 20 and 10% (w/v), premixed PBS buffer (pH 7.4) were supplied by Roche Diagnostics (Mannheim, Germany). Ammonium bicarbonate puriss p.a. (eluent additive for LC-MS) and glycine hydrochloride solution (100 mM glycine in 0.1 M HCl, analytical standard) was supplied by Fluka (Buchs, Switzerland). Tris-HCl Buffer (50 mM pH 8.3) was supplied by Applichem (Darmstadt, Germany). Sitagliptin was purchased from MedChemexpress (Sollentuna, Sweden). Gly-Pro-AMC, recombinant DPP4, L-glutamine, 10 mM HEPES buffer, L-triiodothyronine, 2,2,2-trifluoroethanol (TFEtOH), ascorbic acid, transferrin, insulin, prostaglandin E1, hydrocortisone, and sodium selenite were purchased from Sigma (Buchs, Switzerland). EGM™-Plus BulletKit™ was purchased from Lonza (Basel, Switzerland). RPTEC/TERT1 were supplied by Evercyte GmbH (Vienna, Austria) and HUVEC were supplied by Lonza (Basel, Switzerland). Rabbit plasma was supplied by the animal care unit of F. Hoffmann-La Roche Ltd. (Basel, Switzerland). 2,2,2-Trifluoroacetic acid (TFA) (purity > 99.5%), Gibco fetal calf serum (FCS), Gibco DPBS buffer (pH 7.2), Pierce magnetic beads coated with streptavidin (10 mg/mL) and Chymotrypsin smart digest kit were purchased from Thermo Fisher Scientific (Rheinach, Switzerland), as well as the custom-synthesized reference peptides APIVNAKKDVVNTKMF and IVNAKKDVVNTKMF.

### Incubation of TN-ApoA1 with DPP4

Biotransformation of TN-ApoA1 with DPP4 was assayed in 0.5 mL Protein LoBind tubes at 37 °C. 6.1 µM TN-ApoA1 with incubated with 1 µg/µL recombinant DPP4 in DPBS buffer pH 7.2 with a total reaction volume of 100 µL. At time points 0, 30, 60 and 120 min, 10 µL of incubation solution was removed and mixed with 40ul 0.1% TFA/TFEtOH 95/5 and analysed.

### Incubation of TN-ApoA1 with RPTEC/TERT1 cells and primary HUVEC cells

Primary Renal Proximal Tubule Epithelial Cells immortalized using the human telomerase reverse transcriptase (RPTEC/TERT1) were cultivated in DMEM-Ham’s F-12 (1:1) media supplemented with 2% FCS, 4 mM L-glutamine, 10 mM HEPES buffer, 5 pM triiodothyronine, 10 ng/mL recombinant human EGF, 3.5 g/mL ascorbic acid, 5 µg/mL transferrin, 5 µg/mL insulin, 25 ng/mL prostaglandin E1, 25 ng/mL hydrocortisone, and 8.65 ng/ml sodium selenite. Human umbilical vein endothelial cells (HUVEC) were cultured in EGM^TM^-Plus BulletKit^TM^ medium. RPTEC/TERT1 and HUVEC were seeded into 24-well plate with 1.2 × 10^5^ cells/well in 300 µL medium and were incubated for 3 days at 37 °C and 5% CO_2_. Before start of treatment, the medium was removed and cells were washed with serum free medium and pre-cultured for 4–24 hrs at 37 °C and 5% CO_2_. After pre-culturing, the medium in the wells was removed and replaced with 300 µL medium including 10 µM of TN-ApoA1. The cell density was 10^6^ cells/well. The cells were incubated for 0.5, 4, 24, 48 hrs at 37 °C and 5% CO_2_. The final incubation samples were stored at −80 °C for the subsequent LC-MS/MS analysis.

### Enzyme incubations

DPP4 enzymatic activity was assayed in a 96-well plate at 37 °C. 25 µM substrate Gly-Pro-AMC was mixed with 75 ng/mL recombinant DPP4 or 20 µg cell lysate in 50 mM Tris-HCl buffer pH 8.2 with total reaction volume of 200 µL. 2 µM Sitagliptin was used as DPP4 specific inhibitor. DPP4 activity was determined kinetically by measuring the velocities of AMC release from the substrate at excitation and emission wavelengths of 380 nm and 460 nm with a fluorescence microplate reader (Molecular Devices, San Jose, USA) set 0.25 minute as time interval^17^.

### Enzymatic digest with Chymotrypsin

Enzymatic digest with chymotrypsin was conducted with the SMART Digest Kit Chymotrypsin according to the user manual. In short, 50 µL sample in Tris Buffer was transferred to a PCR tube containing the SMART Digest standard resin slurry and 150 μL digest buffer. Digestion was conducted in a heated shaker at 1100 rpm at 70 °C for 40 min. To stop the digestion, the tubes were centrifuged and 5 μL of the supernatant were directly injected into the HPLC system.

### LC-MS instrumentation

A Thermo Scientific Dionex UltiMate NCP-3200RS Binary Rapid Separation HPLC system was used in combination with a Pal autosampler (CTC Analytics AG, Zwingen, Switzerland) and a Thermo Scientific Orbitrap Fusion Tribrid Mass Spectrometer (Thermo Scientific, Bremen, Germany) equipped with an electrospray ionization (ESI) source. The components of the UltiMate system were specifically designed for operating at pressures up to 800 bar.

### Chromatographic separation of intact proteins

The isolated protein fraction was analyzed on a monolithic HPLC column ProSwift RP from Thermo Fisher Scientific (Reinach, Switzerland), 200 µm i.d. x 250 mm, at a flow rate of 10 µL/min. Mobile Phase A consisted of 0.1% aqueous formic acid, and mobile Phase B of 0.1% aqueous formic acid/acetonitrile 5/95. At the start of the linear gradient, eluent B was kept at 5% for 2 min and then raised to 95% within 14 min and maintained for 2 min, and then decreased again to 5% for re-equilibration of the column.

### Chromatographic separation of peptides from digest with chymotrypsin

A 5 µL aliquot of the sample following solid phase extraction was injected onto the analytical column by gradient elution at a flow rate of 10 µL/min. At the start of the linear gradient, eluent B was kept at 2% for 2 min and then raised to 45% within 30 min. Thereafter, eluent B was increased to 95% and maintained for 3 min, and then decreased again to 2% for re-equilibration of the column. The trapping column and the analytical column were kept at 70 °C during the whole analysis sequence.

## Results

The major *in vivo* biotransformation product of TN-ApoA1 is formed by cleavage of AP from the N-terminus^16^. In this study, formation of the major catabolite [3-285] of TN-ApoA1 by with DPP4, in RPTEC/TERT1 and HUVEC cells was investigated. For this, an optimized analytical method for increased sensitivity based on LC-MS analysis of chymotrypsin-digested TN-ApoA1 was developed.

### LC-MS analysis of chymotrypsin-digested protein

Previous methods were based on tryptic or Lys N digest, resulting in the N-terminal peptides APIVNAK or APIVNA, respectively, for the intact TN-ApoA1 molecule. These peptides could be used to monitor the cleavage of AP by analysis of plasma samples taken at different sampling times. However, the corresponding signature peptides IVNAK or IVNA of the catabolite [3-285] could not be monitored due to its low abundance and poor sensitivity of these two short peptides. For this reason, a method was developed based on chymotrypsin digestion, yielding the signature peptides APIVNAKKDVVNTKMF for TN-ApoA1 and IVNAKKDVVNTKMF for the catabolite [3-285]. Using this refined method, monitoring of the formation of catabolite [3-285] by its key signature peptide IVNAKKDVVNTKMF could be achieved in this study.

### Formation of the major catabolite [3-285] of TN-ApoA1 with DPP4

DPP4, a serine peptidase, cleaves dipeptides from the N-terminus of proteins containing A or P in the amino acid two position, XAX or XPX. In case of TN-ApoA1, the N-terminus motif is API, suggesting cleavage by DPP4. Therefore, the biotransformation of TN-ApoA1 by DPP4 was investigated.

**Figure 1:**
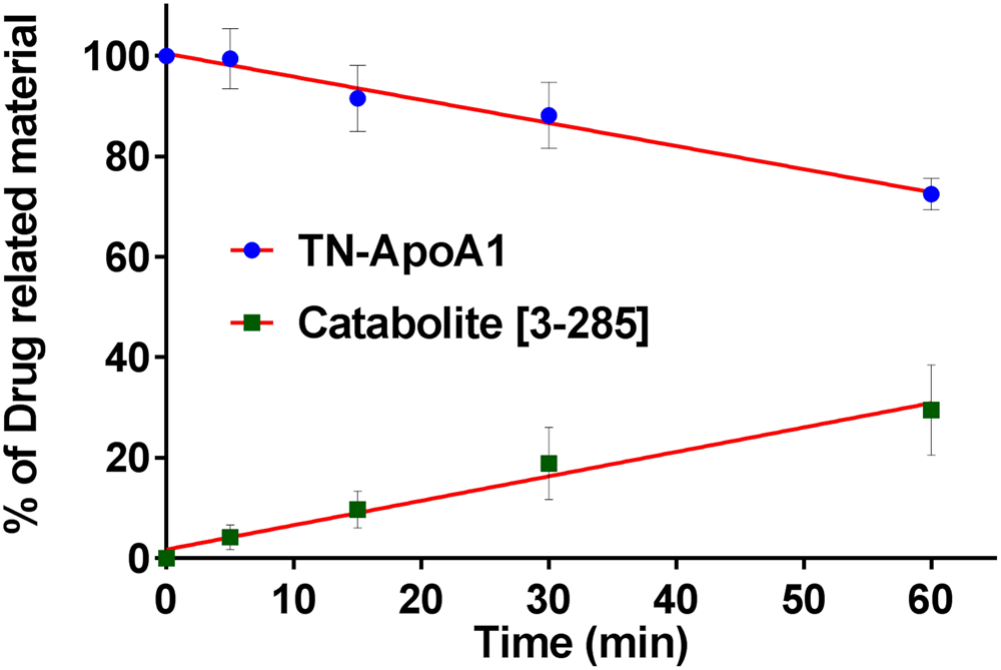
Biotransformation of TN-Apo A1 to [3-285], catalyzed by recombinant DPP4.

After incubation of TN-ApoA1 with DPP4, cleavage of AP to catabolite [3-285] was observed in a time-dependent manner (Fig. 1). Both the disappearance of TN-ApoA1 and the appearance of the catabolite [3-285] could be monitored by intact protein analysis as well as by following specific chymotryptic peptides via LC-MS. In addition, an untargeted search for additional catabolites was performed, but no other catabolites were detected.

Characterization of DPP4 activity in different cell models

**Figure 2:**
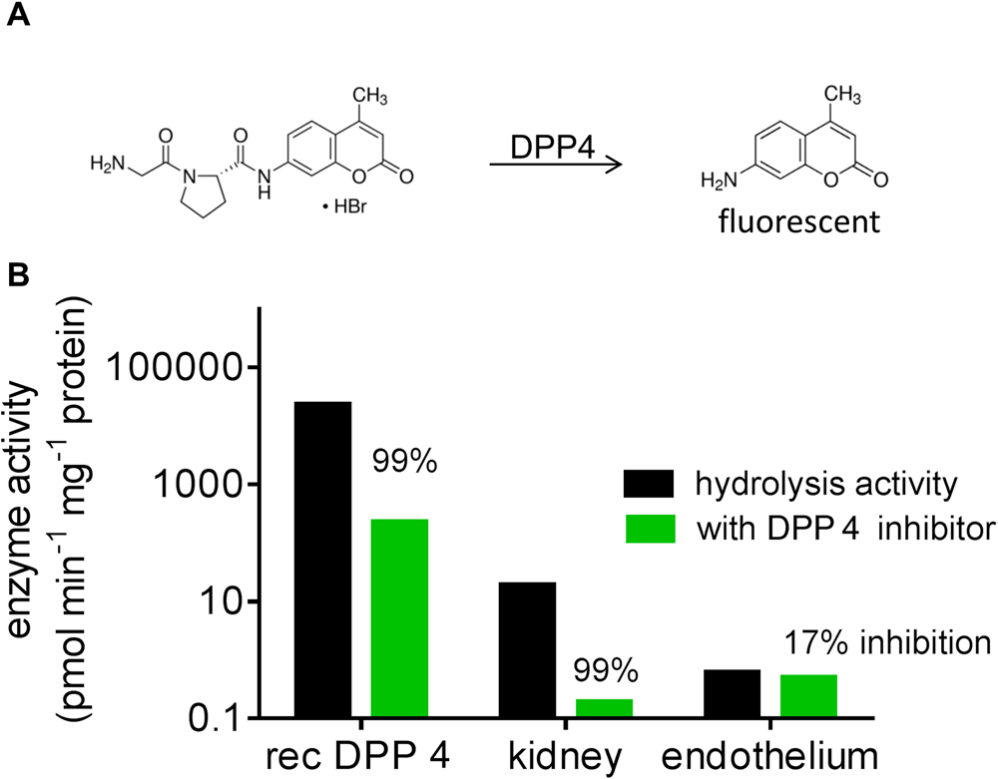
A DPP4 catalyzed enzymatic reaction of Gly-Pro-AMC to AMC. B DPP4 activity of recombinant DPP4, RPTEC/TERT1 and HUVEC and DPP4 inhibition by 1 µM Sitagliptin.

After successful demonstration of TN-ApoA1 cleavage by DPP4 to catabolite [3-285], the next aim was to incubate TN-ApoA1 in cellular *in vitro* models expressing DPP4. Human primary renal proximal tubule epithelial cells (RPTEC/TERT1) and umbilical vein endothelial cells (HUVEC) were investigated as relevant high and low expressing cell lines with respect to DPP4, respectively^18,19^. In a first step, the selected cellular models were characterized for their DPP4 activity. DPP4 activity was assessed using recombinant human DPP4 as a control, Gly-Pro-AMC as substrate and sitagliptin as DPP4 specific inhibitor (Fig. 2). The determined hydrolysis rates are 24.3 µmol/min/mg, 20.2 nmol/min/mg and 0.58 nmol/min/mg for recombinant human DPP4, RPTEC/TERT1 lysate and HUVEC lysate, respectively. RPTEC/TERT1 lysate had about 35-fold higher hydrolysis activity than HUVEC lysate. In the presence of 2 µM sitagliptin, the hydrolysis activity was almost fully inhibited in the incubates of recombinant human DPP4 and RPTEC/TERT1 lysate; but only 17% inhibition of hydrolysis activity was observed with the HUVEC lysate (Fig. 2B). This indicated that DDP4 was the main enzyme involved in the hydrolysis of Gly-Pro-AMC in RPTEC/TERT1; the hydrolysis activity from HUVEC was not predominantly catalyzed by DPP4, and other hydrolysis enzymes rather than DPP4 hydrolyse the substrate Gly-Pro-AMC. K_m_ values were determined for recombinant human DPP4, RPTEC/TERT1 and HUVEC cell lysates with values of, 60 ± 16 µM, 26 ± 2 µM and 47 ± 6 µM determined, respectively (Supplemental Information Fig. 1). The K_m_ value for recombinant DPP4 was in good agreement with that reported by *Matheeussen et al. (*58.8 µM)^17^.

### Formation of the major catabolite [3-285] of TN-ApoA1 by RPTEC/TERT1

**Figure 3:**
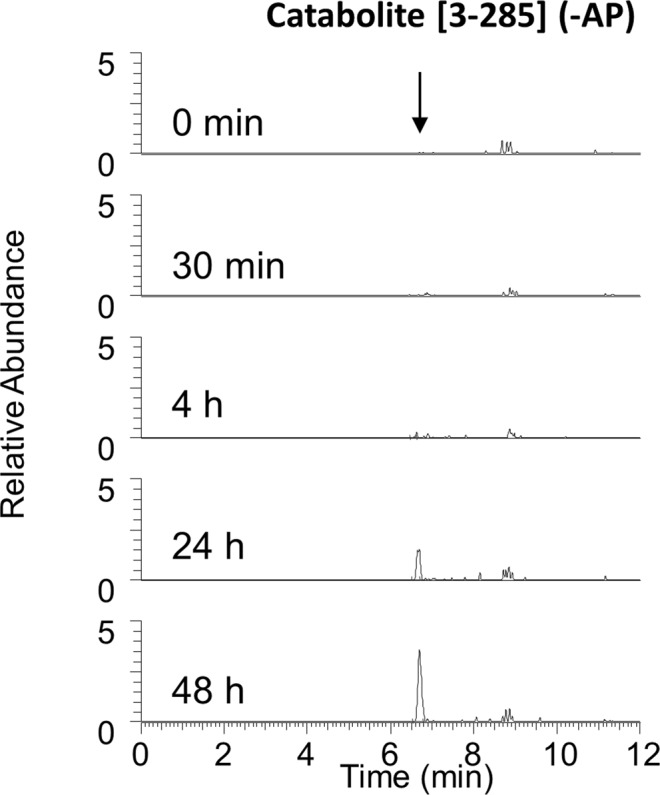
Appearance of catabolite [3-285] that is generated by cleavage of N-terminal aminoacids alanine and proline (AP) in RPTEC/TERT1 via monitoring of chymotryptic signal peptide IVNAKKDVVNTKMF at retention time 6.7 min.

Formation of the major catabolite [3-285] of TN-ApoA1 by RPTEC/TERT1 and HUVEC was similarly investigated using RPTEC/TERT1. After 4 h of incubation the first traces became visible, and the major catabolite [3-285] increased after 24 h and 48 h of incubation, but remained below 5% of TN-ApoA1 (Fig. 3 and Supplemental Figs 2 and 3). When incubated with HUVEC cells, however, biotransformation was too low to be detectable. This is consistent with the 35-fold lower DPP4 activity in HUVEC cells compared to RPTEC/TERT1 cells.

## Discussion

The *in vivo* biotransformation of lipidated TN-ApoA1 has been described previously^16^. The formation of the major biotransformation product [3-285] of TN-ApoA1, generated by cleavage of AP at the N-terminus of the sequence, was fast and complete *in vivo* within 24 h after intravenous administration of TN-ApoA1 to rabbits and also in cynomolgus monkeys and rats. As this biotransformation product is pharmacologically active and therefore conveys most of the pharmacolocigal effect of TN-ApoA1, it is essential to understand the underlying mechanims of this biotransformation. DPP4 was thought to be a likely candidate for TN-ApoA1 cleavage, since the fusion protein contains the typical N-terminal recognition sequence Xaa-Pro-Xaa or Xaa-Ala-Xaa^12^. DPP4 is widely expressed throughout the body, in endothelial cells, including kidney, liver, intestinal brush-border membranes, capillary endothelium, as well as in the vasculature^11^, enabeling it to act as a high capacity biotransformation enzyme for circulating peptides and biologic drugs. The major *in vivo* biotransformation of TN-ApoA1 could be qualitatively reproduced in a cellular system using the renal proximal tubule epithelial kidney cells RPTEC/TERT1, applying a targeted and highly sensitive detection method based on chymotrypsin digest of TN-ApoA1 and its catabolite [3-285]. This catabolite was also formed to some extent *in vitro* in plasma from rabbit, rat, Cynomolgus monkey, and man after incubation at room temperature for 24 h^16^. In HUVEC cells however, where expression of DPP4 is significantly lower than in RPTEC/TERT1, no biotransformation was observed. To our knowledge, this is one of the first reports of an *in vitro* biotransformation study of a therapeutic protein using cellular models. The reason is most likely because it is experimentally challenging to study the *in vitro* biotransformation of therapeutic proteins. The major challenges include low biotransformation rate by cells, also in this present case, even though DPP4 is expressed on the cell surface. However, the low *in vitro* biotransformation rate currently significantly limits the application of *in vitro* biotransformation studies of therapeutic proteins, e.g. for developing predictive clearance tools, or for *in vitro* drug-drug-interaction assessment. One potential solution to increase the sensitivity would be the application of more sensitive analytical methods, like accelerator mass spectrometry or cavity ring down spectroscopy^20^, however these require ^14^C labeling of the therapeutic protein. Another potential refinement of the method could be the use of organ-on-a-chip technology with increased incubation times to provide the necessary sensitivity. Nevertheless the information from such experiments is essential to understand and predict species differences, presence of active catabolites and unexpected clearance as well as performance of bioanalytical assays.

*In vitro* tools for the assessment of mechanisms of biotransformation and other major clearance pathways of therapeutic proteins will enable the estimation of *in vivo* PK properties, elucidate species differences and help rank multiple compounds early on in research in order to put the most promising candidates into development. A mechanistic understanding of the biotransformation of biotherapeutics and responsible proteolytic enzymes is key also for further compound optimization and stabilization against proteolytic cleavage. Although different mechanisms have to be adressed in the context of biologics disposition, the scope is not comparable to the nowadays widely established tools for early characterization of small molecule disposition. Nevertheless, case studies such as this one will help in the development of a wider knowledge-base from which the biotransformation of biotherapeutics can be assessed in future. This is of especial interest where a biotherapeutic agent is substantially converted to an active catabolite with either therapeutic or toxicological implications^16^.

## Supplementary information

Supplementary Information

## Data Availability

The datasets generated during and/or analysed during the current study are available from the corresponding author on reasonable request.
