# Supplementary material for: The In Vitro Biotransformation of the Fusion Protein Tetranectin-Apolipoprotein A1
Source: Sci Rep. 2019 Mar 11;9:4074. doi: 10.1038/s41598-019-40542-5 (PMC6411889; doi:10.1038/s41598-019-40542-5)
Supplement: Supplementary file 1 — Supplementary Information [file 41598_2019_40542_MOESM1_ESM.pdf]

Supplemental Material

The In Vitro Biotransformation of the Fusion Protein Tetranectin-Apolipoprotein A1

Simone Schadt,[1] Christophe Husser,[1] Roland F. Staack,[2] Aynur Ekiciler,[1] Na Hong Qiu,[1] Stephen Fowler,[1] Christoph Funk,[1] Nicole A. Kratochwil[1]

[1] Roche Pharma Research and Early Development, Roche Innovation Center Basel, F. Hoffmann-La Roche Ltd, Grenzacherstr. 124, CH-4070 Basel, Switzerland

[2] Roche Pharma Research and Early Development, Roche Innovation Center Munich, Roche Diagnostics GmbH, Nonnenwald 2, 82377 Penzberg, Germany

Corresponding Author

* Tel: +41 61 687 24 07. E-mail: simone.schadt@roche.com

Cell culturing and subculturing

The RPTEC-TERT1 were culture at 37ºC, 5% CO_2 and 85% humidity until 90% confluence was reached with the DMEM-Ham's F-12 (1:1) supplemented with 4 mM L-glutamine, 10 mM HEPES buffer, 5 pM triiodothyronine, 10 ng/mL recombinant human EGF, 3.5 g/mL ascorbic acid, 5 µg/mL transferrin, 5 µg/mL insulin, 25 ng/mL prostaglandin E1, 25 ng/mL hydrocortisone, 8.65 ng/mL sodium selenite and 100 µg/mL G418, with medium being exchanged every two days. Confluent cultures were washed twice with sterile PBS without Ca_2 and Mg_2 and incubated for 5 min at 37ºC with 2mLof Accutase cell detachment solution. Cell detachment was facilitated by repeated pipette aspirations. As to stop Accutase solution activity, trypsin inhibitor solution was added. The addition of the trypsin inhibitor solution was needed due to the fact that the medium used for this cell line does not contain FBS. The cells plus medium were centrifuged for 5 min at 1200 rpm and the supernatant was discarded. The cells were seeded at a density between $4x10^4$ and $6x10^4$ cells/mL and incubated. 10 mL of fresh, pre-warmed medium was added to the cells, the content was transferred to a t25-flask which was then returned to the incubator.

Thawing of cells

The frozen primary HUVEC Lonza pooled donor were thawed slowly by adding medium into the vial and transferring the thawed content into a tube with 9 mL of EBM-2 Basal medium supplemented with EGM-2 SingleQuot Kit. The cells were then centrifuged for 5 minutes at 1200 rpm. The supernatant was discarded and the cells were transferred to a t25-flask containing 10 mL of fresh medium.

Culturing and subculturing

The cells were culture at 37ºC, 5% CO_2 and 85% humidity, exchanging media until confluence was reached. Medium exchange was performed by warming up fresh culture medium at 37°C in water bath for at least 30 min. The used medium was carefully poured from the t-flask into a waste pot. Immediately, 10 mL of fresh pre-warmed culture medium was added to the t-flask which was then returned to the incubator. Confluent cultures were washed twice with sterile PBS without Ca_2 and Mg_2 and incubated for 5 minutes at 37ºC with 2 mL of Accutase cell detachment solution. Cell detachment was facilitated by repeated pipette aspirations. As to stop Accutase solution activity, the cells were transferred to 10 mL of fresh medium and centrifuged for 5 min at 1200 rpm. The supernatant was discarded and fresh medium was added. Cells were seeded at a density between $4x10_5$ and $8x10^5$cells/mL and incubated.

Preparation of cell lysates

As to measure the DPP-IV levels in RPTEC/TERT1 and HUVEC cells, 1 mL of each which contained 10^6 cells was collected from the culture medium and centrifuged at 13000 rpm for 5 min. The medium was discarded and the cells were washed twice by adding 1mL of PBS to the cell pellet, mixing thoroughly and centrifuging at 13000 rpm for 5 min. The solution was discarded and replaced with 1 mL of 50 mM Tris-HCl buffer pH 8.3 containing 0.1% TritonX. The cells were lysed which was performed by vortexing and keeping the cells 1 h on ice or at 4°C to ensure the cells were properly lysed they were vortext in between as well. Until use the cell lysates were keept at -20ºC.

Enzyme incubations

DPPIV enzymatic activity was assayed in a 96-well plate at 37°C. 25 µM substrate Gly-Pro-AMC were mixed with 75 ng/mL recombinant DPPIV or 20 µg cell lysate in 50 mM Tris-HCl buffer pH 8.2 with total reaction volume of 200 µL. 2 µM Sitagliptin was used as DPPIV specific inhibitor. DPPIV activity was determined kinetically by measuring the velocities of AMC release from the substrate at wavelengths λex of 380 nm and λem of 460 nm with a Fluorescence Microplate Reader (Molecular Devices, San Jose, USA) set 0.25 minute as time interval (Matheeussen et al., 2012).

Enzymatic digest with Chymotrypsin

Enzymatic digest with chymotrypsin was conducted with the SMART Digest Kit Chymotrypsin according to the user manual. In short, 50µl sample in Tris Buffer was transferred to a PCR tube containing the SMART Digest standard resin slurry and 150 µL digest buffer. Digestion was conducted in a heated shaker at 1100 rpm at 70°C for 40 min. To stop the digestion, the tubes were centrifuged and 5 µL of the supernatant were directly injected into the HPLC system.

Characterization of DPP4 activity

Gly-Pro-AMC hydrolysis in recombinant human DPP4

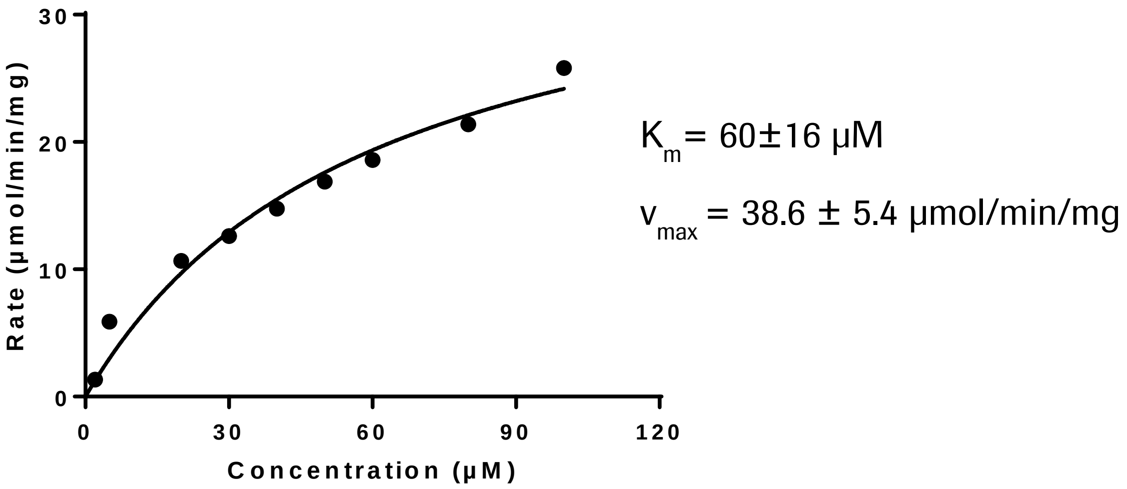

Gly-Pro-AMC hydrolysis in RPTEC/TERT1 lysate

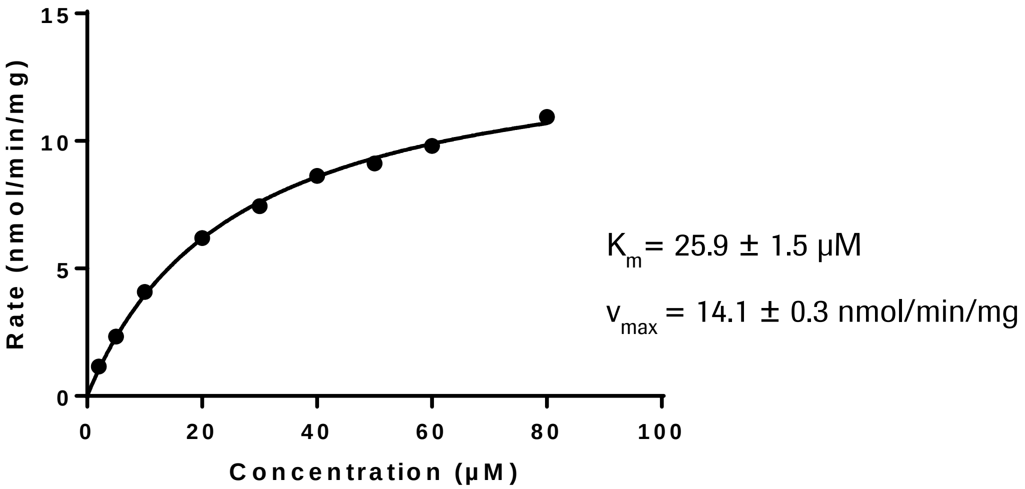

Supplemental Figure 1: K_m values were determined for recombinant human DPP4, RPTEC/TERT1 and HUVEC, and were in a similar range (60 ± 16 µM for recombinant DPP4, 26 ± 2 µM for RPTEC/TERT1 lysate and 47 ± 6 µM for HUVEC lysate respectively.

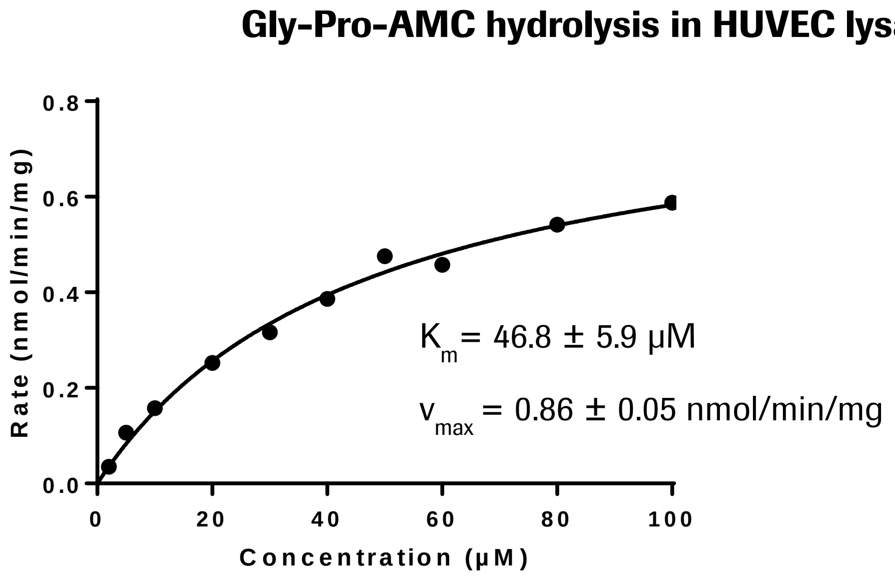

Supplemental Figure 1 (continued): K_m values were determined for recombinant human DPP4, RPTEC/TERT1 and HUVEC, and were in a similar range (60 ± 16 µM for recombinant DPP4, 26 ± 2 µM for RPTEC/TERT1 lysate and 47 ± 6 µM for HUVEC lysate respectively.

Biotransformation of TN-ApoA1 by DPP4

incubation time, min	TN-ApoA1, %				Average, %	Stdev
0	100	100	100	100	100	
5	93.7	104.4	105.0	95.1	99.5	6.0
15	83.8	93.8	99.5	89.4	91.6	6.6
30	84.4	92.8	94.6	81.0	88.2	6.6
60	68.0	74.3	74.8	73.1	72.5	3.1

incubation time, min	catabolite [3-285], %				Average, %	Stdev
0						
5	5.3	7.1	3.0	1.5	4.2	2.5
15	11.0	12.4	11.2	4.2	9.7	3.7
30	21.6	22.8	22.9	8.2	18.9	7.5
60	31.6	32.9	37.0	16.4	29.5	9.0

Biotransformation of TN-ApoA1 by RPTEC/TERT1

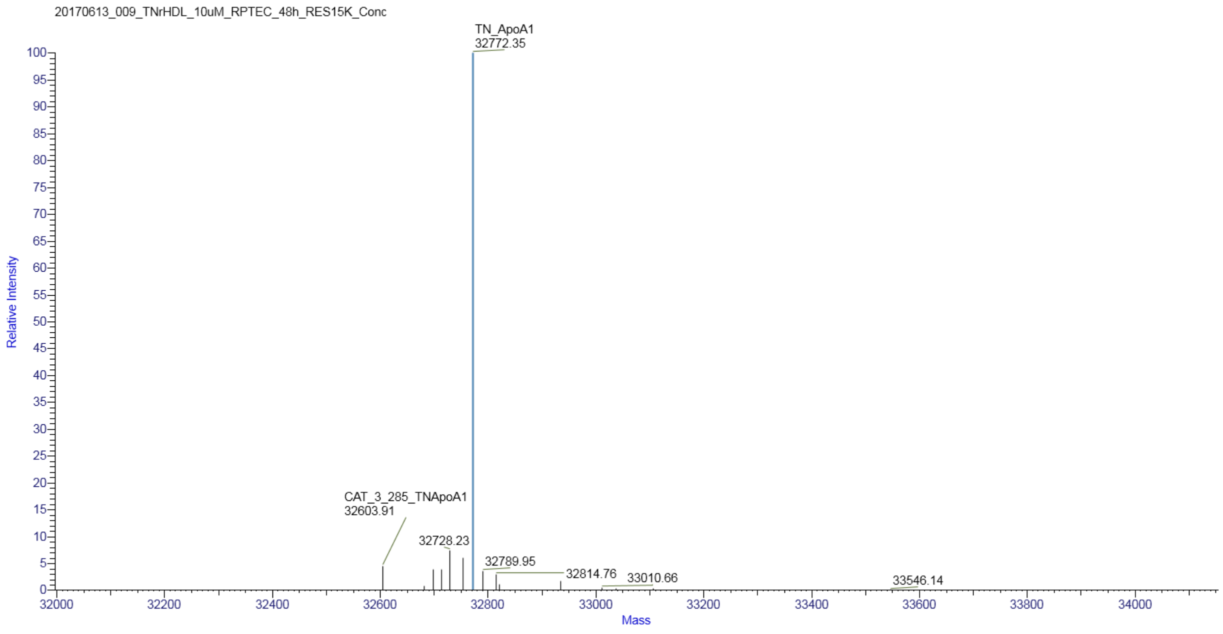

Supplemental Figure 2: Deconvoluted spectrum after incubation of TN-ApoA1 in RPTEC/TERT1 cells for 48 h. In addition to TN-ApoA1 (m/z 32772), a signal of the catabolite [3-285] (m/z 32604) can be seen in the spectrum.

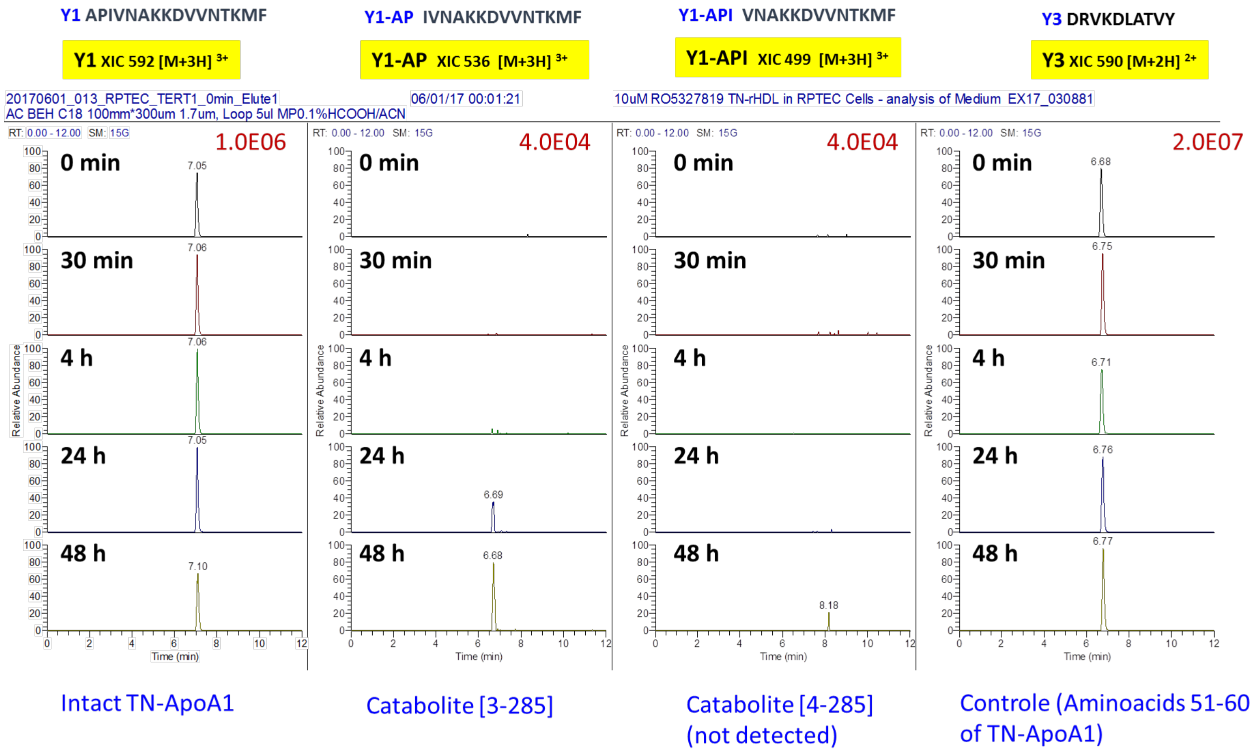

Supplemental Figure 3: Various chymotryptic signal peptides after incubation of TN-ApoA1 in RPTEC/TERT1 cells for 0 min, 30 min, 4 h, 24 h, and 48 h (from top to bottom). From left to right: signal peptide APIVNAKKDVVNTKMF for TN-ApoA1, signal peptide IVNAKKDVVNTKMF for Catabolite [3-285], signal peptide VNAKKDVVNTKMF for Catabolite [4-285] (not detected in this experiment for any time point, low signals in chromatograms are background noise and not specific), signal peptide DRVKDLATVY in the middle of the protein (controle). The peak intensity corresponding to 100 % of the chromatograms in arbitrary units is indicated by the red numbers on upper right of the four different signal peptide time course chromatograms.
